# Ultrasound-Guided Suprainguinal Versus Infrainguinal Fascia Iliaca Compartment Block for Postoperative Analgesia After Total Knee Replacement: A Prospective Randomized Trial

**DOI:** 10.7759/cureus.84453

**Published:** 2025-05-20

**Authors:** Vinod K Srivastava, Adarsh K Singh, Neel K Mishra, Rati Prabha, Rajesh Raman, Vinita Singh, Shailendra Singh

**Affiliations:** 1 Anesthesiology, King George's Medical University, Lucknow, IND; 2 Orthopedic Surgery, King George's Medical University, Lucknow, IND

**Keywords:** acute pain management, fascia iliaca compartment block, postoperative pain, spinal anesthesia, total knee replacement, ultrasound-guided regional anesthesia

## Abstract

Background: Fascia iliaca compartment block (FICB) can be achieved using suprainguinal (SFICB) or infrainguinal (IFICB) approaches. This study compares postoperative analgesia of SFICB and IFICB after total knee replacement (TKR).

Methods: This prospective, randomized, single-blind study was conducted on 60 adult patients undergoing unilateral TKR under spinal anesthesia. Patients were randomly allocated into two groups: Group S received SFICB with 30 mL of 0.25% levobupivacaine with 2 mL (8 mg) dexamethasone, while Group I received IFICB with the same drugs after surgery. The primary outcome variable was 24-hour cumulative tramadol consumption. Secondary outcome variables included pain intensity, complications, hemodynamic variations, and time to first rescue analgesic request.

Results: Tramadol consumption was significantly lower with SFICB (86.67±34.57 versus 34.57±33.95 mg, p=0.006). Pain intensity was lower with SFICB after six hours of surgery. Time to first analgesic request was also longer with SFICB (12.83±3.80 versus 10.12±2.98 hours, p=0.003). Time taken for administering the blocks (16.20±2.66 versus 17.17±3.13 minutes), complications, and hemodynamic variations were statistically similar between the groups.

Conclusion: SFICB provides superior postoperative analgesia compared to IFICB in patients undergoing TKR, with reduced opioid requirement and prolonged analgesia duration.

## Introduction

Total knee replacement (TKR) is a widely performed orthopedic surgery intended to restore joint function and relieve pain in patients suffering from end-stage knee osteoarthritis. Inadequate pain control can impair mobilization, prolong hospital stay, and negatively impact overall patient recovery and satisfaction [1,2]. Regional anesthesia techniques play a pivotal role in the multimodal analgesic regimen used in postoperative analgesia of these patients. These techniques allow for localized pain control with minimal systemic effects. Among the various options, fascia iliaca compartment block (FICB) has gained attention for its ability to provide effective analgesia after TKR. FICB anesthetizes the femoral nerve, lateral femoral cutaneous nerve (LFCN), and sometimes the obturator nerve (ON) by injecting local anesthetic beneath the fascia iliaca [3-5]. These nerves provide sensory innervation to the knee.

Two techniques exist for FICB administration: the traditional infrainguinal (IFICB) approach and the more recently described suprainguinal (SFICB) method. IFICB involves needle insertion below the inguinal ligament. While IFICB is a well-established technique, studies have demonstrated that its spread of injected drug may be suboptimal, particularly in achieving consistent block of the LFCN and ON. In contrast, the SFICB involves injecting the local anesthetic above the inguinal ligament. This causes more cephalad spread and greater efficacy in blocking the femoral nerve, LFCN, and ON. This potentially enhances the analgesic efficacy of SFICB [3]. Clinically, this may translate to superior analgesia, especially in surgeries where the obturator nerve's contribution to pain is significant, such as TKR.

However, literature comparing these two techniques for postoperative analgesia, particularly in the context of TKR, is limited. Most existing studies have focused on hip surgery or general lower limb trauma, with limited data directly assessing their application in knee arthroplasty. Our trial tries to address this gap by comparing the efficacy of SFICB versus IFICB. This trial aims to compare the postoperative analgesia of IFICB and SFICB for patients undergoing TKR. We hypothesized that the SFICB, by virtue of its more proximal injection site and potential for better anesthetic spread, would result in longer-lasting analgesia, less pain intensity, and decreased requirement of additional analgesics compared to IFICB. The primary objective was to compare ultrasound-guided suprainguinal and infrainguinal approaches of the FICB for postoperative analgesic consumption for patients undergoing TKR. Secondary objectives were to compare the two approaches of FICB for postoperative pain intensity, time taken for block execution, time to first analgesic request, and complications in postoperative patients after TKR.

## Materials and methods

This prospective, single-blind, randomized, comparative clinical trial was conducted at a tertiary care teaching hospital. Ethical clearance was obtained from the Institutional Ethics Committee of King George's Medical University, Lucknow (approval number: 1395/Ethics/2023), and the trial was registered with the Clinical Trials Registry of India (CTRI/2024/04/065691). Patient recruitment was done between April 25, 2024, and December 16, 2024. Informed written consent was obtained from all participants after a thorough explanation of the study procedure, risks, and benefits. The trial included patients aged between 18 and 65 years, of either gender, belonging to American Society of Anesthesiologists physical status I or II, scheduled for elective TKR under spinal anesthesia. We excluded patients with inability to comprehend the visual analog scale (VAS), contraindications to regional anesthesia, and refusal to give consent.

Eligible patients were randomly allocated to two equal groups (n=30 each) using a computer-generated randomization sequence with sealed opaque envelopes to ensure allocation concealment. Group S received the SFICB, while Group I received the traditional IFICB. After the arrival of the patients in the operating room, anesthesia monitors (pulse oximeter, non-invasive blood pressure, and electrocardiogram) were applied to the patient. All patients were administered intrathecal anesthesia using 3 mL of 0.5% hyperbaric bupivacaine combined with 25 μg fentanyl in the L3-L4 interspace under standard aseptic precautions, and the surgery was started. The patients were blinded to the study group. Group allocation was revealed only to the anesthesiologists after the surgical procedure was completed. The assigned block was administered postoperatively by the anesthesiologist with at least three-year experience in administering the blocks used in the study. For the IFICB (Group I), the patients were positioned supine. After skin preparation and under sterile conditions, a high-frequency linear ultrasound probe (6-13 MHz) was placed in a transverse orientation just distal to the inguinal ligament. The femoral artery superficial to the fascia iliaca and the femoral nerve deep to the fascia iliaca were identified. A 22-G, 8-cm echogenic nerve block needle was advanced in-plane from lateral to medial beneath the fascia iliaca. Once the correct plane was confirmed by hydrodissection with 2 mL saline, 30 mL of 0.25% levobupivacaine with 2 mL (8 mg) dexamethasone was administered in incremental doses after negative aspiration to avoid intravascular injection. For the SFICB (Group S), the blocks were given in a similar supine position. The ultrasound probe was placed perpendicular to the inguinal ligament and then moved cranially until the "bowtie appearance" formed by the anterior inferior iliac spine, iliacus, and sartorius was obtained. The deep circumflex iliac artery was used as a key landmark. The block needle was inserted in-plane from the lateral direction beneath the fascia iliaca and cephalad to the inguinal ligament. Hydrodissection was used to confirm correct needle tip placement, followed by the injection of the same drug mixture in a similar incremental fashion [3].

All recruited patients were given 1 g of intravenous paracetamol every eight hours postoperatively. Intravenous tramadol 50-100 mg was administered as a rescue analgesic when the VAS score was ≥4 or when the patient requested analgesia for pain, up to a maximum of 300 mg within 24 hours. The dose of tramadol was 100 mg for VAS pain intensity 7-10 and 50 mg for VAS pain intensity ≤6. The primary outcome was the cumulative dose of tramadol administered to the patients in the initial 24 hours after surgery. Secondary outcome variables were postoperative VAS, time to first analgesic request, time taken to administer the block, complications (including, but not limited to, postoperative nausea and vomiting and hematoma) due to the block, and hemodynamic variables (mean arterial pressure and heart rate) change. Pain intensity and hemodynamic changes were assessed at one, two, four, six, 12, 18, and 24 postoperative hours.

Statistical analysis was done using SPSS version 27.0 (IBM Corp., Armonk, NY). Continuous variables are expressed as mean ± standard deviation and compared using the unpaired t-test. Ordinal data are expressed using median with interquartile range and were compared using the Mann-Whitney U test. Categorical variables were analyzed using Fisher's exact test. A p-value of less than 0.05 was considered statistically significant. The sample size was calculated with a power of 0.8 and a type I error of 0.05 using the SPSS software. A previous trial studying the two approaches of FICB had rescue analgesic consumption of 68.75±51.2 and 34.38±30.1 mg [6]. Using these values of rescue analgesic consumption, a minimum of 25 patients were needed in each group. To compensate for data loss and patient exclusions, 30 patients were included in each group.

## Results

**Figure 1:**
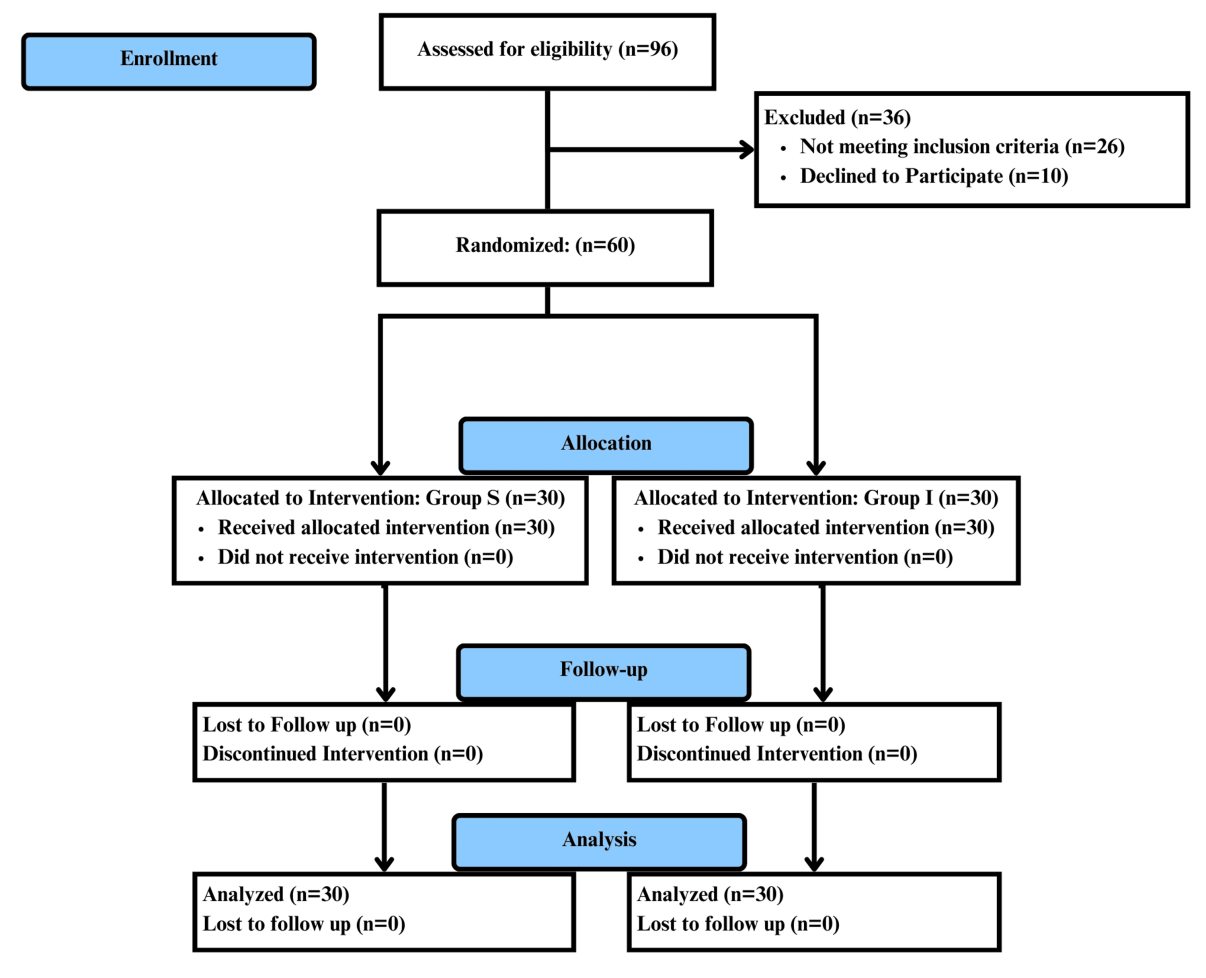
Patient flowchart for the trial

**Figure 2:**
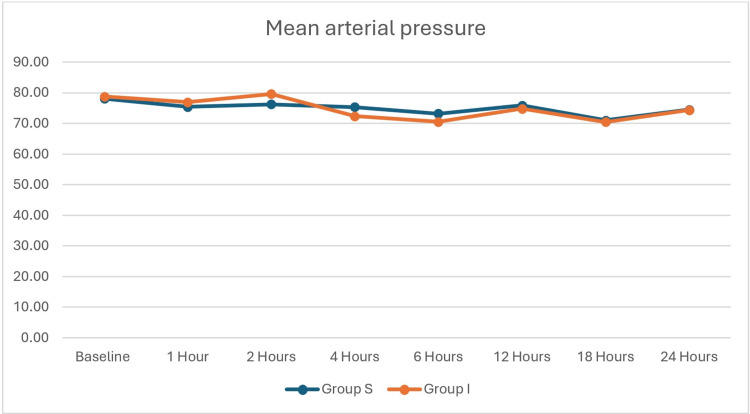
Mean arterial pressure (in mmHg) of the patients

**Figure 3:**
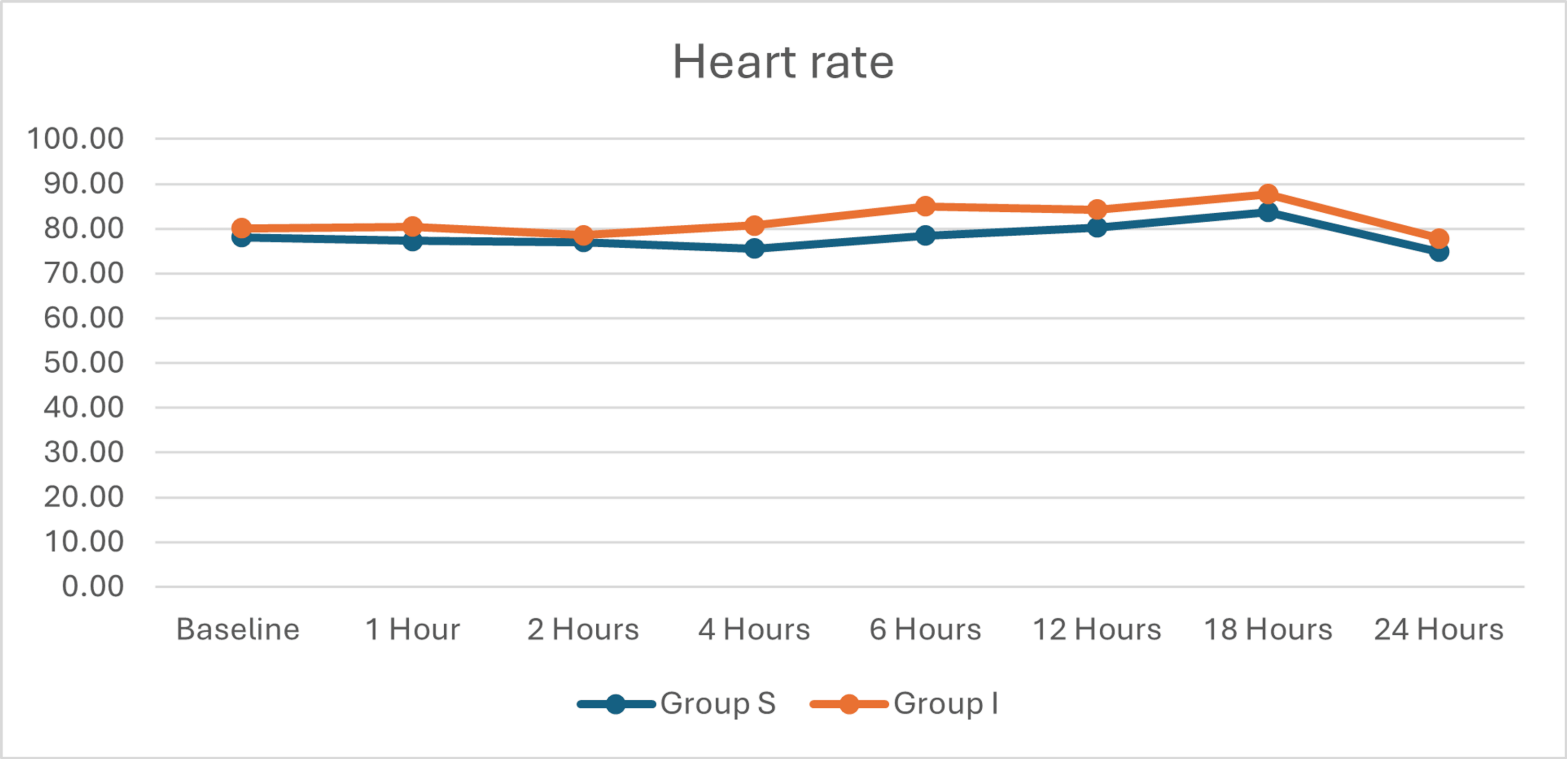
Heart rate (in beats per minute) of the patients

**Figure 4:**
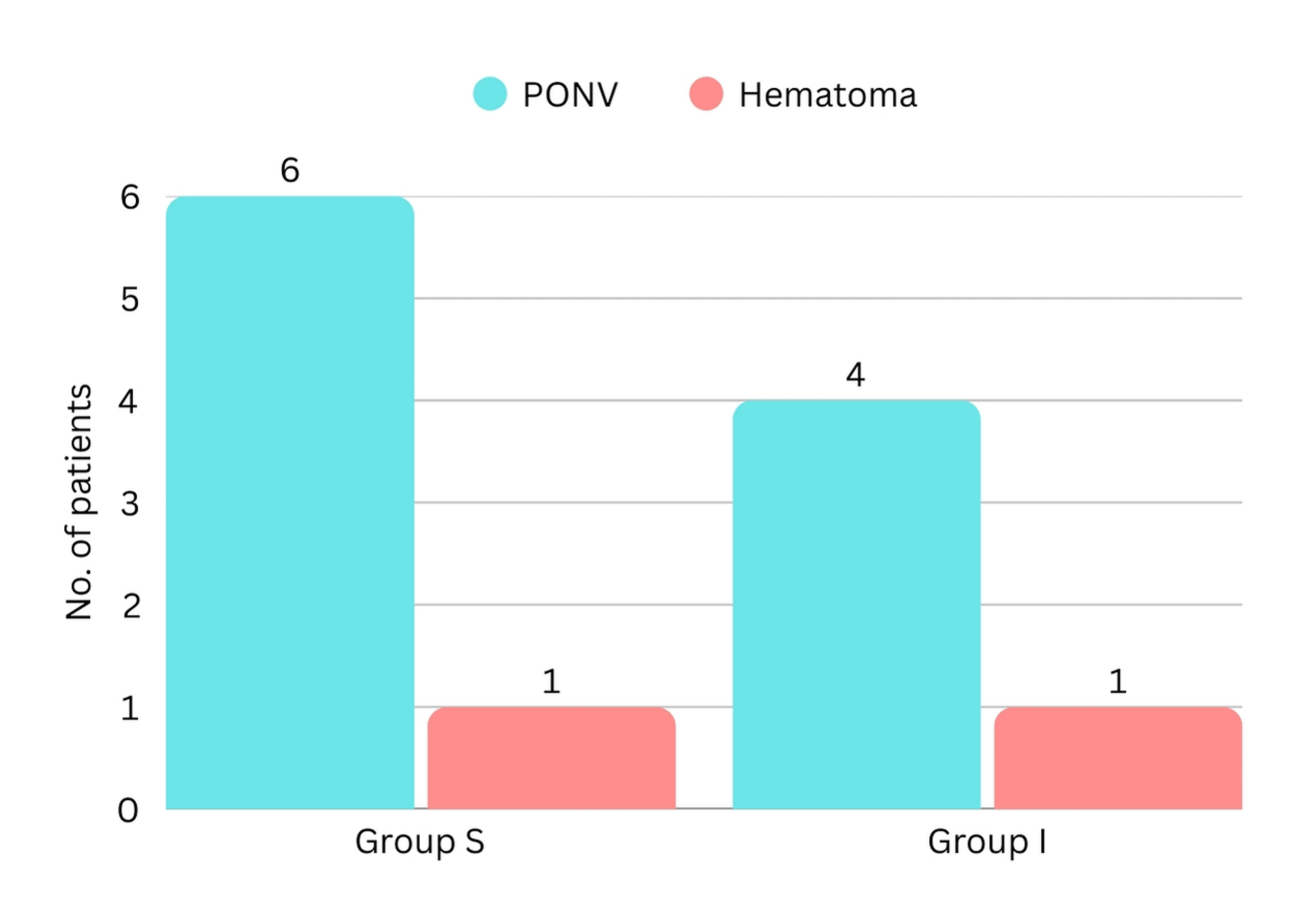
Frequency of complications in the two groups PONV: postoperative nausea and vomiting

The flowchart of the patient selection process is shown in Figure 1. All continuous data, except VAS, were evenly distributed. The baseline and demographic data of the participants in the two groups are shown in Table 1. These were statistically similar. Time taken for administering the block was statistically similar, as shown in Table 2. However, the time (12.83±3.80 versus 10.12±2.98 hours, p=0.003) for the first analgesic request was statistically longer and the rescue analgesic dose was statistically lower in Group S. Postoperative pain is compared in Table 3. The postoperative pain was statistically similar until the first six postoperative hours. However, postoperative pain was statistically lower in Group S at 12, 18, and 24 postoperative hours. Hemodynamic variables (Figure 2 and Figure 3) were statistically similar between the groups. Complications are shown in Figure 4. Six patients in Group S and four in Group I had postoperative nausea and vomiting. One patient in each group had a local hematoma. These were statistically similar.

**Table 1:** Baseline characteristics of the patients BMI: body mass index, ASA: American Society of Anesthesiologists physical status classification, CI: confidence interval, NA: not applicable

Characteristics	Group S (n=30)	95% CI	Group I (n=30)	95% CI	Test statistic	p
Age (years)	52.63±9.81	48.97-56.30	54.70±10.28	50.86-58.54	t=0.797	0.429
Height (cm)	165.93±2.83	164.88-166.99	164.63±4.30	163.03-166.24	t=1.385	0.171
Weight (kg)	67.33±5.36	65.33-69.34	64.97±7.27	62.25-67.68	t=0.042	0.157
BMI (kg/m^2^)	24.45±1.76	23.79-25.11	23.95±2.38	23.06-24.84	t=0.923	0.360
Duration of surgery	154.73±34.33	141.91-167.55	141.63±33.21	129.23-154.03	t=1.502	0.138
Gender (male/female)	11 (36.7%) / 19 (63.3%)	NA	14 (46.7%) / 16 (53.3%)	NA	χ^2^=0.617	0.601
ASA (I/II)	21 (70.0%) / 9 (30.0%)	NA	20 (66.7%) / 10 (33.3%)	NA	χ^2^=0.077	1.000

**Table 2:** Block characteristics of the patients *: statistically significant, CI: confidence interval

Characteristics	Group S (n=30)	95% CI	Group I (n=30)	95% CI	Test statistic	p
Time taken for block (minutes)	16.20±2.66	14.86-16.48	17.17±3.13	16.00-18.34	t=2.157	0.202
Time for first analgesic request (hours)	12.83±3.80	11.42-14.25	10.12±2.98	9.00-11.23	t=0.187	0.003*
Rescue dose (tramadol in mg)	86.67±34.57	73.76-99.58	111.67±33.95	98.99-124.34	t=0.792	0.006*

**Table 3:** Postoperative pain of the patients VAS: visual analog scale, *: statistically significant

VAS	Group S (n=30)	Group I (n=30)	Test statistic	p
1 hour	1 (1-1)	1 (1-1)	U=450.00	1.000
2 hours	1 (1-1)	1 (1-1)	U=450.00	1.000
4 hours	2 (2-3)	3 (2-3)	U=395.00	0.348
6 hours	2.5 (2-3)	3 (1-3)	U=420.00	0.731
12 hours	2.5 (2-4)	4 (3-4)	U=195.00	<0.001*
18 hours	3 (2-5)	3.5 (3-5)	U=199.50	<0.001*
24 hours	3 (1.25-5)	4 (3-5)	U=146.00	<0.001*

## Discussion

This study was conducted to compare the postoperative analgesia of SFICB and IFICB for patients undergoing TKR. It was observed in our study that SFICB was superior to IFICB in providing analgesia after TKR. The postoperative tramadol consumption and pain on the VAS were lower and the duration of analgesia was longer in patients receiving SFICB. Optimal pain control in TKR is important, as it contributes significantly to early mobilization, reduction in hospital stay, and overall improvement in patient satisfaction and functional recovery [1]. The FICB was introduced as a technique to achieve blockade of the femoral nerve, LFCN, and ON by directing the drug solution into the fascial plane beneath the fascia iliaca [5]. However, its efficacy has been reported to be variable, possibly due to inconsistent spread of the local anesthetic, particularly with the infrainguinal approach. However, the drug injection in the SFICB technique involves cephalad injection of the local anesthetic medication. This results in a more cephalad spread of the injected drugs and consistent blockade of ON. Several recent studies have supported our findings that the suprainguinal approach results in improved analgesic outcomes in patients undergoing lower limb surgeries [6-8].

The main findings of our study demonstrate that the SFICB group experienced significantly better postoperative pain control compared to the IFICB group. This was evidenced by a lower cumulative dose of opioid consumption in the first 24 postoperative hours. SFICB and IFICB were compared for postoperative analgesic efficacy after TKR in a previous study [7]. The authors recruited adult patients undergoing TKR for this randomized prospective study. As in our study, it was concluded by the authors that total postoperative opioid consumption was significantly less in patients with SFICB. The authors also studied the blockade of the ON by the two approaches using electromyography. It was observed by the authors that SFICB blocked the ON in a significantly higher proportion of patients. Anatomically, the suprainguinal approach enables more proximal deposition of the local anesthetic, leading to a more effective blockade of the LFCN and ON. The ON and LFCN are less frequently blocked by IFICB. The more consistent spread of anesthetic drug to the LFCN and ON accounts for the superior analgesic outcomes observed with SFICB [7,9,10]. In a study conducted on 10 volunteers, SFICB produced a block of the ON in eight out of 10 volunteers. IFICB produced a block of ON in one out of the 10 patients [9]. The infrainguinal technique, while still effective, may not consistently anesthetize all the targeted nerves, leading to inadequate analgesia in patients. The reduction in opioid use has important implications given the well-documented adverse effects of opioids, such as vomiting, ileus, thromboembolism, urinary retention, and respiratory depression [11-13].

The suprainguinal group had significantly lower VAS after the sixth postoperative hour. We could not find studies comparing the two block approaches in patients undergoing TKR. However, for hip and femur surgeries, patients receiving SFICB had lower pain scores [6,8]. This finding is similar to the observations in the current study. This can be explained by the favorable spread of local anesthetics with SFICB. This anatomical advantage translates clinically into a consistent sensory blockade, particularly of the obturator nerve, which is often missed with the infrainguinal approach but contributes significantly to knee joint innervation. Our study extends these findings to the context of knee arthroplasty.

The time taken for the first rescue analgesia was statistically higher in the SFICB (12.83 versus 10.12 hours), indicating a prolonged duration of effective analgesia. In previous studies involving lower limb surgeries, SIFCB has provided a longer duration of analgesia, which is consistent with the findings of the study [6,14,15]. This finding is clinically important because early and sustained analgesia facilitates participation in physiotherapy and early ambulation, key components in successful recovery after TKR. Various authors have emphasized regional anesthesia techniques that minimize systemic opioid use and support rapid functional recovery [2,11,12]. Our findings clearly demonstrate that the SFICB offers superior analgesic efficacy over the IFICB. These findings support the incorporation of SFICB into routine clinical practice for TKR patients. The findings of our study carry significant implications for clinical practice.

The complications (nausea and vomiting, and hematoma) in the two groups were statistically similar. The complications were infrequent, minor, and easily treatable. In addition, hemodynamic parameters remained stable post-block in both groups, highlighting the safety profile of both techniques.

One of the key strengths of this study is its focus on a specific and clinically relevant surgical population: patients undergoing total knee replacement. Previous studies have primarily explored SFICB in proximal femur fractures, hip fractures, or hip replacement surgeries. Our results provide evidence for expanding the use of SFICB to knee surgeries. The limitations of the study include the single-center design of the study, which may negatively impact the generalization of the study. The single-blind design of the study may have introduced observer bias as the anesthetist performing the block and recording the data was aware of the group allocation of the patients. This is the second limitation. Another limitation was the administration of the blocks by a single anesthetist, which may limit the generalizability of the study. Also, the follow-up period was limited to only 24 hours. Therefore, variables such as long-term pain control and late-onset complications were not studied. Lastly, the study did not assess functional outcomes, such as mobilization time or patient satisfaction, which are relevant to regional anesthesia efficacy.

## Conclusions

In conclusion, our study demonstrates that SFICB provides superior postoperative analgesia compared to IFICB after TKR. Compared to IFICB, SFICB reduces postoperative tramadol use, lowers postoperative pain intensity, and prolongs analgesia duration. SFICB is equivalent to IFICB in terms of adverse events. The effectiveness of SFICB makes it a valuable tool for anesthesiologists managing postoperative pain after TKR. Larger multicentric trials are warranted to confirm the study findings across different demographic and clinical settings.
