# Correction: Ultrasound-Guided Suprainguinal Versus Infrainguinal Fascia Iliaca Compartment Block for Postoperative Analgesia After Total Knee Replacement: A Prospective Randomized Trial

**DOI:** 10.7759/cureus.c228

**Published:** 2025-07-07

**Authors:** Vinod K Srivastava, Adarsh K Singh, Neel K Mishra, Rati Prabha, Rajesh Raman, Vinita Singh, Shailendra Singh

**Affiliations:** 1 Anesthesiology, King George's Medical University, Lucknow, IND; 2 Orthopedic Surgery, King George's Medical University, Lucknow, IND

This article has been corrected to fix a typo in Table 1. The Pearson Chi-square value and the p value for the test are correctly reported in Table 1 (Pearson Chi-square value =0.077, p= 1.000). However, the number of patients belonging to ASA I and ASA II in group S were mistakenly interchanged while preparing the manuscript. The article mentions that for group S, ASA I had 9 (30.0%) and ASA II had 21 (70.0%) patients. This has been corrected to state that group S had 21 (70.0%) ASA I patients and 9 (30.0%) ASA II patients. Implications and interpretations of the test are correctly described in the article and do not require any further corrections.

